# Renal hypouricemia caused by novel compound heterozygous mutations in the *SLC22A12* gene: a case report with literature review

**DOI:** 10.1186/s12881-018-0595-8

**Published:** 2018-08-10

**Authors:** Zhaowei Zhou, Lidan Ma, Juan Zhou, Zhijian Song, Jinmai Zhang, Ke Wang, Boyu Chen, Dun Pan, Zhiqiang Li, Changgui Li, Yongyong Shi

**Affiliations:** 1 Bio-X Institutes, Key Laboratory for the Genetics of Developmental and Neuropsychiatric Disorders (Ministry of Education), Shanghai Jiao Tong University, No. 1954 Huashan Road, Shanghai, 200030 People’s Republic of China; 2 Shandong Gout Clinical Medical Center, Qingdao, 266003 People’s Republic of China; 3 Shandong Provincial Key Laboratory of Metabolic Disease, The Affiliated Hospital of Qingdao University, No. 16 Jiangsu Road, Qingdao, 266003 People’s Republic of China; 4 The Department of Endocrinology and Metabolism, The Affiliated Hospital of Qingdao University, Qingdao, 266003 People’s Republic of China; 5 Biomedical Sciences Institute, the Qingdao Branch of SJTU Bio-X Institutes, Qingdao University, Qingdao, 266003 People’s Republic of China; 6 Metabolic Disease Institute, Qingdao University, Qingdao, 266003 People’s Republic of China

**Keywords:** Hypouricemia, Chinese, *SLC22A12*, Mutation, Whole-exome sequencing

## Abstract

**Background:**

Renal hypouricemia (RHUC) is a heterogeneous genetic disorder that is characterized by decreased serum uric acid concentration and increased fractional excretion of uric acid. Previous reports have revealed many functional mutations in two urate transporter genes, *SLC22A12* and/or *SLC2A*9, to be the causative genetic factors of this disorder. However, there are still unresolved patients, suggesting the existence of other causal genes or new mutations. Here, we report an RHUC patient with novel compound heterozygous mutations in the *SLC22A12* gene.

**Case presentation:**

A 27-year-old female presenting with recurrent hypouricemia during routine checkups was referred to our hospital. After obtaining the patient’s consent, both the patient and her healthy parents were analyzed using whole-exome sequencing (WES) and Sanger sequencing to discover and validate causal mutations, respectively. The prioritization protocol of WES screened out two mutations of c.269G > A/p.R90H and c.1289_1290insGG/p.M430fsX466, which are both located in the *SLC22A12* gene, in the patient. Sanger sequencing further confirmed that the patient’s heterozygous c.269G > A/p.R90H mutation, which has been reported previously, derived from her mother, and the heterozygous c.1289_1290insGG/p.M430fsX466 mutation, which was found for the first time, derived from her father. p.R90H, which is highly conserved among different species, may decrease the stability of this domain and was considered to be almost damaging in silicon analysis. p.M430fsX466 lacks the last three transmembrane domains, including the tripeptide motif (S/T)XΦ (X = any amino acid and Φ = hydrophobic residue), at the C-terminal, which interact with scaffolding protein PDZK1 and thus will possibly lead to weak functioning of urate transport through the disruption of the “transporter complex” that is formed by URAT1 and PDZK1.

**Conclusions:**

We report a Chinese patient with RHUC, which was caused by compound heterozygous mutations of the *SLC22A12* gene, using WES and Sanger sequencing for the first time. Mutation-induced structural instability or malfunction of the urate transporter complex may be the main mechanisms for this hereditary disorder.

**Electronic supplementary material:**

The online version of this article (10.1186/s12881-018-0595-8) contains supplementary material, which is available to authorized users.

## Background

Renal hypouricemia (RHUC) is a relatively rare heterogeneous genetic disorder that is characterized by decreased serum uric acid concentration (SUA < 2.0 mg/dl) and increased fractional excretion of uric acid (FEUA > 10%), which itself does not cause evident discomfort, but it may lead to severe complications, such as urolithiasis and acute kidney injury (AKI), after strenuous exercise in some patients [1]. The confirmation diagnosis and specific subtyping are determined by molecular analysis of the *SLC22A12* gene (encoding uric acid transporter 1, URAT1) [2] and the *SLC2A9* gene (encoding glucose transporter member 9, GLUT9) [3]. URAT1 was the first identified renal urate-anion transporter that is mainly expressed in the apical side of the proximal tubule, and three homozygous loss-of-function mutations were confirmed to cause idiopathic renal hypouricemia (classified as RHUC1) [2]. Until now, dozens of mutations in the *SLC22A12* gene (24 missense mutations [2, 4–12], 2 nonsense mutations [2, 4], 3 small deletions [4, 11, 12], 1 splice-site mutation [4], and 1 gross deletion mutation [5]) have been detected and functionally characterized in more than 150 RHUC patients from all over the world, but mainly in Japan. GLUT9 is a voltage-driven urate efflux transporter from tubular cells into blood and the interstitium and its loss-of-function mutation was first found in a non-*SLC22A12* defect RHUC patient (classified as RHUC2) [3]. The detected mutations in the *SLC2A9* gene were fewer but more pathogenic in inducing severe manifestations compared with that of *SLC22A12*. Genome-wide association studies (GWASs) aim to identify disease/phenotype-associated susceptibility genes and multiple GWASs have correspondingly confirmed a strong correlation of both *SLC2A9* and *SLC22A12* with SUA concentration [10, 13–20], providing further evidence of their causative role in RHUC. In addition, many transporters have been postulated to be involved with urate flux pathways in the renal proximal tubule: OAT4/*SLC22A11* and OAT10/*SLC22A13* function as apical urate reabsorption transporters in the exchange of organic anions; OAT1/*SLC22A6*, OAT2/*SLC22A7*, and OAT3/*SLC22A8* are located in the basolateral membrane and translocate urate from the interstitium into the tubule coupled with α-ketoglutarate (α-KG) or other unknown anions exiting, and then urate is secreted by apical transporters of MRP4/*ABCC4*, ABCG2, NPT1/*SLC17A1*, and NPT4/*SLC17A3* [21]. Further, a scaffolding protein of PDZ domain containing 1 (PDZK1) was identified as modulating urate transport by interacting with a complex of URAT1, OAT4, NPT1, and ABCG2 [22, 23]. Previous reports on RHUC mainly focused on the molecular analysis of the well-confirmed causal genes *SLC22A12* and/or *SLC2A9*, and there was still a handful of patients with an absence of defects [4, 6, 7, 24, 25], indicating the complexity of urate homeostasis and the existence of other causal genes and mutations. There is no consensus on the treatment for RHUC and outcomes may be severe [26], which raises the urgency of an early and accurate diagnosis for the early prevention of urolithiasis and AKI. Our study, for the first time, comprehensively applied whole-exome sequencing (WES) technology and Sanger sequencing to identify the causal genes and mutations in an autosomal recessive family of an RHUC daughter and healthy parents.

## Case presentation

### Case report

The index patient was a 27-year-old Chinese Han female with non consanguineous parents. She was accidentally noted to have a low SUA level on two occasions (SUA = 0.52 mg/dl and 0.45 mg/dl) in her routine check-up in the local hospital. She was asymptomatic and had no other discomfort, such as joint pain, loin pain, hematuria, urine foam, nausea, vomiting, anorexia, or diarrhea. No special treatment was recommended. She was recently admitted into the metabolism division, the Affiliated Hospital of Qingdao University due to the repeated detection of hypouricemia; thorough laboratory and imaging examinations were carried out. Biochemical tests confirmed the diagnosis of hypouricemia (SUA = 0.33 mg/dl) with hyperuricosuria (FEUA = 50%). The current reference ranges for SUA used in our laboratory department are 3.5–7.0 mg/dL in males and 2.5–6.0 mg/dL in females. Meanwhile, the reference range for FEUA is 4–10%, irrespective of gender. Other biomedical findings were within normal range or negative. No kidney stones were found by local ultrasound. Acquired hypouricemia resulting from Fanconi syndrome and other clinical disorders were excluded. Both parents of the patient are alive and healthy, with no evident medical history, such as urolithiasis and AKI. The SUA concentration in the father was 3.68 mg/dl, which was within the lower-normal range, and the mother was 4.35 mg/dl, which was within the fully normal range. A FEUA test was not performed in both parents because they refused to have it done. The patient does not have any siblings. There is no history of hereditary diseases and there are no specific physical abnormalities. In addition, the patient stated she was a vegetarian who did not favor meat. Based on the overall information, we concluded that the patient had idiopathic RHUC. Clinical data are shown in Table 1. Pharmacologic therapy was not administered due to the absence of significant clinical manifestations and the lack of guidelines for RHUC management. However, several medical orders were required in order to inhibit the urate overproduction and prevent the incidence of adverse events: the patient was instructed to drink plenty of water and consume a low purine diet, avoid engaging in strenuous exercise, alkalize urine with sodium bicarbonate continuously and moderately, and monitor renal function regularly via laboratory and imaging examinations during subsequent outpatient follow-up. The case reports (CARE) guidelines were followed.

**Table 1:** Clinical data and confirmed *SLC22A12* mutations in patient and both parents

Individuals	Gender	Age	SUA(mg/dl)	FEUA(%)	Clinical symptoms	*SLC22A12* mutations		State
						nucleotide	amino acid	
Patient	Female	27y	0.33	50	–	c.269G > A/c.1289_1290insGG	p.R90H/p.M430fsX466	compound heterozygote
Father	Male	NA	3.68	NA	–	c.1289_1290insGG/WT	p.M430fsX466/WT	heterozygote
mother	Female	NA	4.35	NA	–	c.269G > A/WT	p.R90H/WT	heterozygote

- denotes negative, *NA* denotes not available

### Genomic DNA preparation

The patient and both of her parents were recruited for this study. Peripheral blood samples were collected upon the patient signing the relevant informed consent. Genomic DNA was subsequently extracted using LifeFeng Genomic DNA Purification Kit (Lifefeng Biotech Co., Ltd., Shanghai, China) and then quantified for concentration and purity evaluation by a NanoDrop 1000 Spectrophotometer (Thermo Scientific, USA). All of the samples were within the reference range (double-stranded DNA concentration: > 20 ng/ul, 260/280: > 1.8, 260/230: > 2.0) that satisfied the requirements of the genomic analysis.

The study was approved by the Ethics Committee of Shanghai Jiao Tong University and the Affiliated Hospital of Qingdao University, and it was conducted in accordance with the principles of the Declaration of Helsinki.

### WES

An initiation of 0.2 μg genomic DNA was subjected to WES. Genomic DNA was sheared into fragments of ~ 150 bp in size by a Covaris™ S2 Ultrasonicator System (Covaris, Woburn, MA, USA) and the product was ligated to paired-end adapters containing Illumina-specific indexes using a SureSelect XT Library Prep Kit ILM (Agilent Technologies, Santa Clara, CA). The resulting product was amplified by ligation-mediated polymerase chain reaction (LM-PCR). The adapter-ligated DNA library was enriched and captured with a SureSelect Target Enrichment Kit Box 1 (Agilent Technologies, Santa Clara, CA). Each well-constructed library was then sequenced as 150 bp paired-end reads on an Illumina HiSeq 2500 platform (Illumina, San Diego, California, USA), at the coverage depth of 50×. Briefly, crude sequencing data were checked for quality control by FastQC v 0.11.2 (http://www.bioinformatics.babraham.ac.uk/projects/fastqc/) and then trimmed by Trimmomatic v 0.30 to exclude unqualified reads. The resulting clean reads were aligned to NCBI build GRCh37/hg19 by Burrows-Wheeler Aligner (BWA) v 0.7.7. The aligned reads were processed to remove the duplications by Picard v 1.110. Then, local realignment and annotation with RefSeq genes, dbSNP 138, and 1000 Genomes Project were performed by Genome Analysis Toolkit (GATK) v 2.8–1 and Annovar software [27], respectively.

### Sanger sequencing

Candidate mutations of interest were further validated by PCR amplification and direct Sanger sequencing. PCR primers were designed using a primer designing tool (http://www.ncbi.nlm.nih.gov/tools/primer-blast/index.cgi?LINK_LOC=NcbiHomeAd) and they were ordered from Shanghai Generay Biotech Co., Ltd., Shanghai, China. Primer sequences are listed in Table 2. PCR amplification was performed on a GeneAmp PCR System 9700 (Applied Biosystems, Foster City, CA) with 2X Taq PCR Master Mix (Lifefeng Biotech Co., Ltd., Shanghai, China), and the product was loaded on ABI 3100 instruments (Applied Biosystems, Foster City, CA) at Shanghai Jieli Biotechnology Co., Ltd. for Sanger sequencing.

**Table 2:** Primer sequence for *SLC22A12* mutations

Primer name	Forward primer sequence	Reverse primer sequence
269 primer	TCCAGGTTCTCCAGACGATG	TCCCAGGACTGGACCTTTGAG
1289 primer	AAACGGGGTCAAGAAGGACTC	CACAAGAGGGAGATGCATGA

### Causality predictions of identified mutations

Annotations with SIFT, Polyphen-2, LRT, and MutationTaster by Annovar, were performed to evaluate the mutation pathogenicity. Since the crystal structure has not been available, TMpred online software and Pymol 2.0, a free and user-friendly molecular visualization tool, were used to predict the secondary functional domains of the URAT1 protein and its 3-D image, respectively.

### Mutations analysis

To screen out the genetic mutations leading to RHUC, WES was applied in genomic DNA from the RHUC patient and both of her parents. On average, the total number of bases in the reads was 4520 Mb and the ratio of bases having a phred score of greater than 30 (Q30) reached up to 94%. The detailed outputs are shown in Table 3.

**Table 3:** Sequencing outputs in patient and both parents

Individuals	Reads	Total base (Mbases)	Q30 (%)
Patient	28,517,742	4278	94.48
Father	29,321,578	4398	94.30
mother	32,628,210	4894	94.38

According to an assumed inheritance of the autosomal recessive mode for this disease, we first analyzed the putative genetic mutations homozygous in the patient and heterozygous in both of the parents. Seeking functional variants, we filtered out those referred to as either unknown or synonymous. Focusing on rare variants, we sought for those having frequencies < 0.01 or unknown in 6500 Exome Sequencing Project database. Then, we excluded those present in the in-house exome sequence data obtained from 16 normal unrelated individuals, and finally, 29 variants were retained (see Sheet 1 in Additional file 1). Unfortunately, none of the variants in the genes were functionally candidates for purine/uric acid metabolism and excretion. Next, we analyzed the putative causal mutations present in the patient but not present in the parents based on the prioritization strategy mentioned above. A total of 521 suspicious mutations were screened out (see Sheet 2 in Additional file 1). In terms of the known candidate genes related to purine/uric acid metabolism and excretion, we finally chose the mutations located in the *SLC22A12* gene for further Sanger sequencing.

**Fig. 1:**
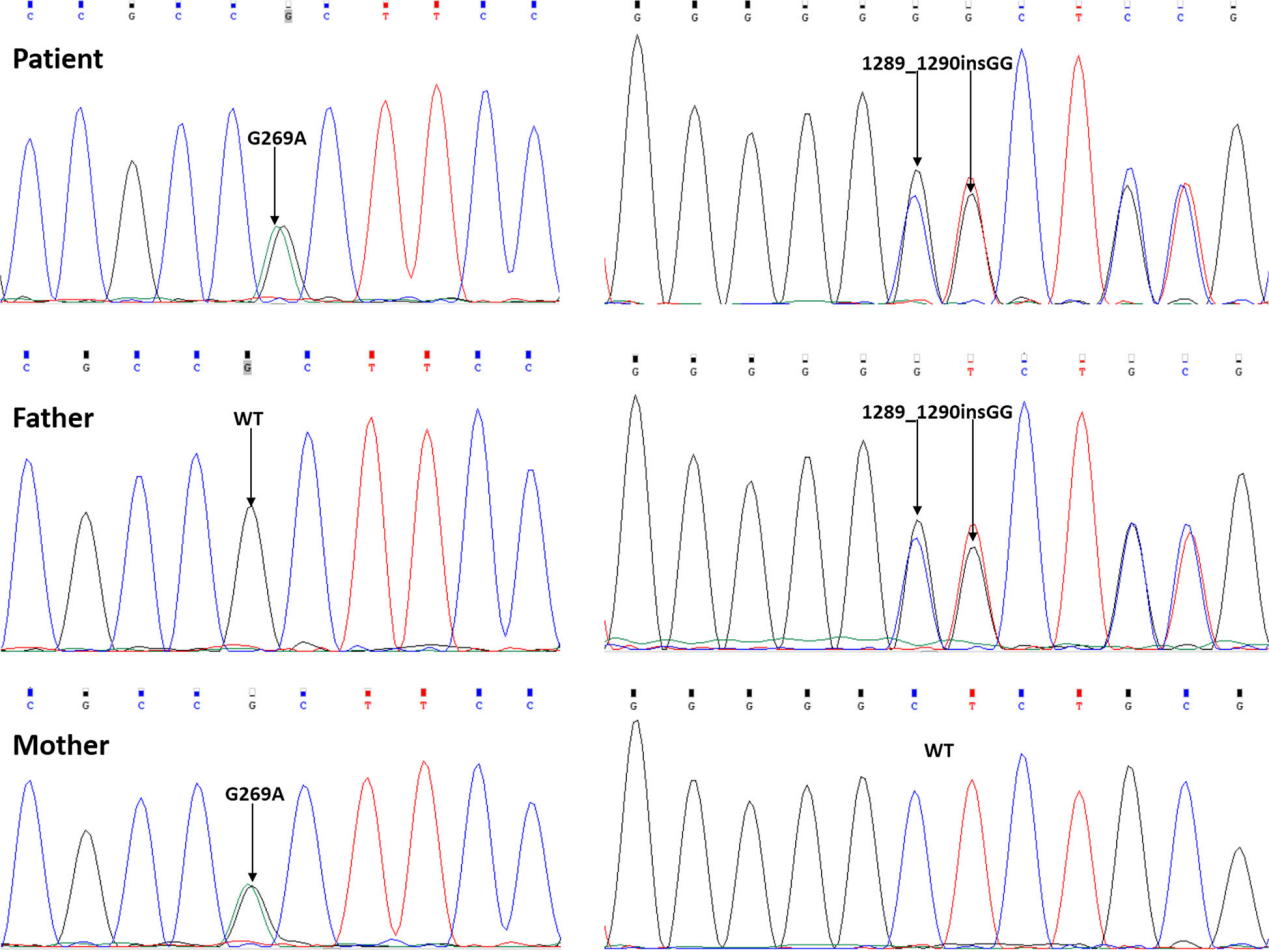
Sanger sequencing for the two mutations in this family. The patient carried two heterogeneous mutations: c.G269A/WT and c.1289_1290insGG/WT. The father carried one heterozygous mutation of c.1289_1290insGG/WT, while the mother carried another heterozygous mutation of c.G269A/WT

Sanger sequencing revealed that the patient possessed two heterozygous mutations (see Table 1 and Fig. 1). One was a G to A substitution at nucleotide 269 in exon 1 (c.269G > A) leading to an alteration of arginine by histidine at codon 90 (p.R90H), which had been confirmed many times [4, 5, 7, 28–32]. Another was an insertion of GG at nucleotide 1289_1290 in exon 8 (c.1289_1290insGG), resulting in a frameshift and a truncated protein of 466 amino acids with a premature stop codon (p.M430fsX466), which was first reported in our study. The heterozygous c.269G > A was verified in the patient’s mother and the heterozygous c.1289_1290insGG in her father, suggesting that each heterozygous mutation of the patient derived from her mother and father, respectively (see Table 1 and Fig. 1).

**Fig. 2:**
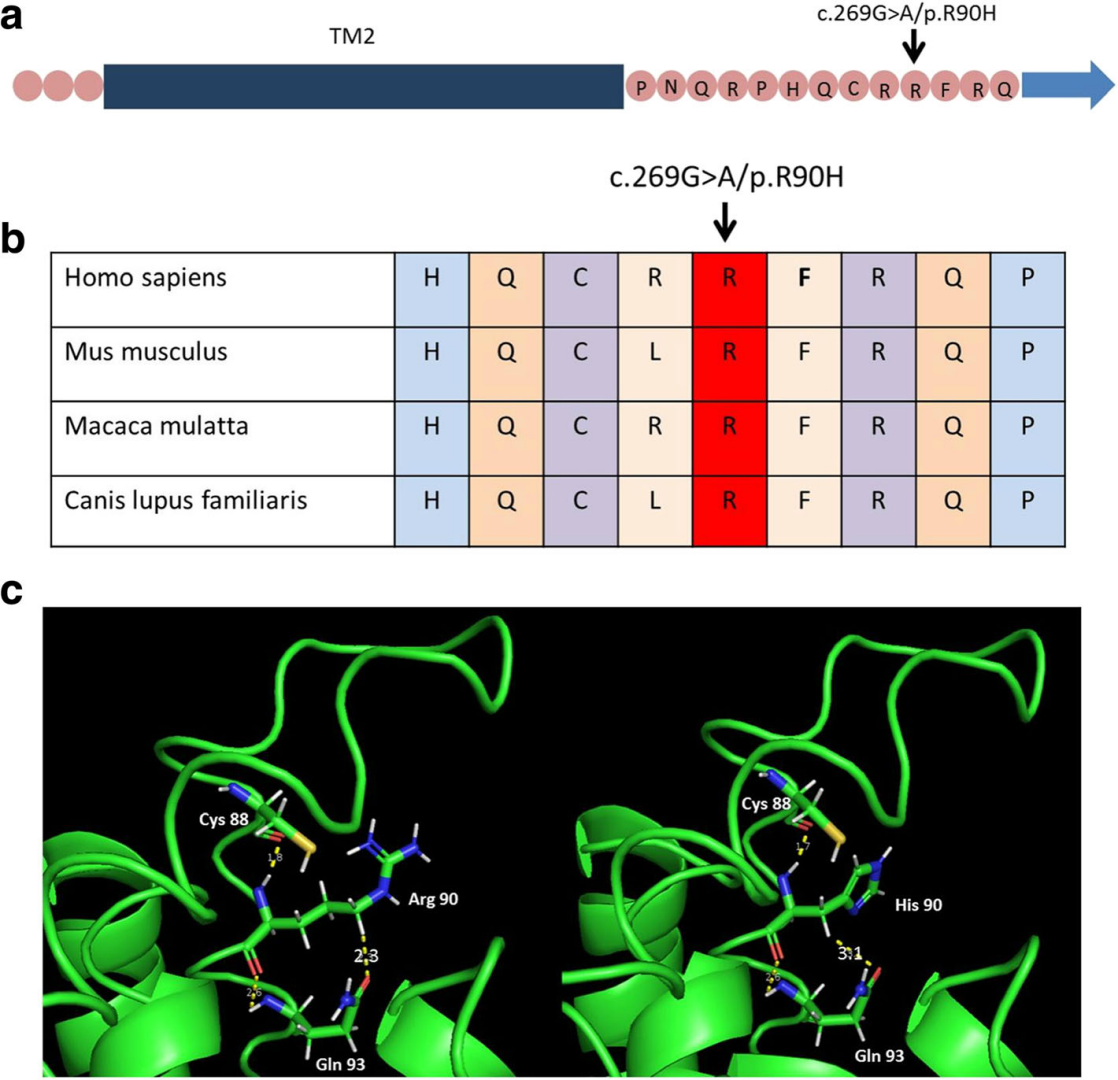
c.269G > A/p.R90H mutation identified in the RHUC patient. **a** This mutation is located close to the second transmembrane region (TM2) as indicated by the black arrow. **b** This identified amino acid substitution displays high evolutionary conservation among different species as indicated by the black arrow. **c** 3-D structures of the wild-type and c.269G > A/p.R90H URAT1 proteins with prediction software. The left shows the structure of the wild type. The right shows the structure of p.R90H. The hydrogen bond between His90 and Gln93 in the mutant is weakly formed (bond length is 3.1 Å) in comparison with the wild type (bond length is 2.3 Å)

The c.269G > A mutation is considered to be “damaging” by Polyphen-2 (= 0.992), LRT (= 0) and MutationTaster prediction (= 1), while it is “tolerated” by SIFT (= 0.061). Protein domain analysis showed that URAT1 has 12 transmembrane (TM) helices, and we made a discovery that amino acid substitution p.R90H, which is located in the intracellular region close to the second TM (TM2) (Fig. 2a), is highly conserved among different species (Fig. 2b). Further, we conducted molecular modeling to predict the effect of this mutation on protein structure (Fig. 2c). In the wild model, Arg90 formed hydrogen bonds with Cys88 and Gln93 effectively, building the conformational stability of this intracellular domain. In the mutation model, the hydrogen bond between His90 and Gln93 were weakly formed, which may decrease the stability of this domain and further affect urate transport activity. The insertion mutation c.1289_1290insGG produced a truncated protein of 466 amino acids from the position 430, with the amino acids sequence altered compared to the wild mature protein of 553 amino acids. Protein domain analysis showed that this truncated protein lacks the last three transmembrane domains, including the tripeptide motif (S/T)XΦ (X = any amino acid and Φ = hydrophobic residue) at the C-terminal, which interacts with scaffolding protein PDZK1, and together they strengthen urate transport [22].

## Discussion and conclusions

Our study confirmed the comprehensive analysis of WES technology in combination with Sanger sequencing as an effective strategy to detect the causal genes and mutations in an autosomal recessive family of an RHUC daughter and healthy parents. The patient was shown to carry two heterozygous loss-of-function mutations of c.269G > A and c.1289_1290insGG, with each allele deriving from her mother and father, respectively.

Despite the well-confirmed causal genes (*SLC22A12* and *SLC2A9*) [2, 3] and other candidate genes related to purine metabolism/urate transport [21, 33], there are still unresolved cases, which suggests still-undiscovered complicity genes and pathogenic mutations. The traditional candidate gene approach only considers a small number of genes; this strategy will fail to detect genetic variants in some patients, especially when the genetic etiology of the disease is not well known. Next-generation sequencing technologies, such as whole genome sequencing and WES, will undoubtedly detect additional genetic variants in an unbiased manner on the genome-wide level compared with traditional candidate gene sequencing. Since we sought to examine variants expected to interrupt protein function (generally located in the coding regions) with relatively lower costs, we selected WES as the prioritized method.

Until now, 31 mutations in the *SLC22A12* gene (24 missense mutations [2, 4–12], 2 nonsense mutations [2, 4], 3 small deletions [4, 11, 12], 1 splice-site mutation [4], and 1 gross deletion mutation [5]) have been detected and functionally characterized in more than 150 RHUC patients all over the world, but most of the patients mainly live in Japan (obtained from http://www.hgmd.cf.ac.uk/ac/all.php). Here, we summarized all of the *SLC22A12* gene mutations for RHUC patients, and their clinical features in varied races were mainly obtained from typical case reports (see Table 4). Among these mutations, the W258X frequency was unexpectedly high with 184 alleles identified in 231 affected alleles (79.7%), comparable to the single study by Ichida et al. (74.1%) [4]. It is noteworthy that RHUC patients harboring at least one W258X mutation were more likely to be experience urolithiasis, AKI, or hematuria, with the proportion of 31.0% (36/116, see Table 4). More strikingly, the ratio reached up to 44.1% in those with a homozygous mutation of W258X (30/68, see Table 4). Combined with the finding of its high frequency in the general population (2.37 and 2.24%) [5, 34], we argue that the W258X mutation is the most prevalent and pathogenic in RHUC patients of Japanese origin. A similar situation occurs in Korea: the W258X allele accounted for 81.8% of all of the affected alleles (9/11, see Table 4), and severe complications were present in 66.7% of the W258X carriers (4/6, see Table 4). These results indicate that the W258X mutation possibly originated on the Asian continent. However, this mutation was not found in Chinese RHUC patients, which may be explained by the small number of reported RHUC patients in China (8 individuals, including those in the present study, see Table 4) and the consequent difficulty in detecting a specific mutation. The R90H mutation ranked as the second prevalent, with the allele frequency being 7.3% (17/231), 9.1% (1/11), and 38.5% (5/13) in Japanese, Koreans, and Chinese with RHUC (see Table 4). Therefore, we suspect that the R90H mutation may be the major contribution to Chinese RHUC patients. However, these two mutations were not found in other races, including Czech Roma, Iraqi Jews, and individuals of European descent (see Table 4). The L415_G417del mutation and R406C mutation were detected in most RHUC patients from the Czech Republic and Iraq, respectively (see Table 4). Recently, a molecular epidemiological study showed that the frequencies of the L415_G417del and T467 M mutations were 1.92 and 5.56%, respectively, in a subgroup of the Roma population from Slovakia, the Czech Republic, and Spain [35]. Thus, we suppose each is the founder of the mutation in their respective populations. We also noted that the mutations in individuals of European descent presented with larger heterogeneity (see Table 4). On the whole, most RHUC patients were carriers of homozygous or compound heterozygous mutations (50.3%, 87/173 and 27.2%, 45/173, respectively; see Table 4) in the *SLC22A12* gene, which may be explained by the whole protein dysfunction. Those individuals with simple heterozygous mutations also accounted for a substantial proportion (18.5%, 32/173) and presented with comparable clinical manifestations (see Table 4), which seemed to differ from the stereotypical interpretation that partial reservation of the normal protein may ameliorate the severity of RHUC. Considering that these patients only had one gene sequenced, the existence of additional causative genes may not be excluded, which can be corroborated by two patients with concurrent mutations in both *SLC22A12* and *SLC2A9* [11].

**Table 4:** Summary of all *SLC22A12* gene mutations and clinical data for RHUC patients in varied races (mainly from case reports)

Races	Nucleotide change^a^	Amino acid change^b^	No. of patients reported	No. of urolithiasis, AKI and hematuria	References
Japan	G774A/G774A	W258X/W258X	68	5, 25, 0	[2, 4–7, 32, 36–44]
	G774A/+	W258X/+	15	1, 1, 0	[4, 6, 7, 29, 37, 44, 45]
	G774A/G269A	W258X/R90H	13	0, 0, 0	[4, 5, 7, 29, 32]
	G774A/1639-1643delGTCCT	W258X/frameshift	4	0, 0, 0	[4, 6, 7]
	G269A/G269A	R90H/R90H	1	0, 0, 0	[7]
	G774A/G412A	W258X/V138 M	3	2, 0, 0	[4, 7]
	G774A/C650T	W258X/T217 M	2	0, 0, 0	[4, 37]
	G774A/C889T	W258X/Q297X	2	0, 1, 0	[4, 32]
	G774A/IVS2 + 1G > A	W258X/Frameshift	2	0, 0, 0	[4, 46]
	G774A/A1145T	W258X/Q382L	2	0, 0, 0	[4, 6]
	G774A/G1082 T	W258X/G361 V	2	0, 1, 0	[7]
	G269A/C889T	R90H/Q297X	1	0, 1, 0	[31]
	G269A/C1429A	R90H/R477S	1	0, 0, 0	[7]
	C650T/C650T	T217 M/T217 M	1	–	[2]
	G894 T/G894 T	E298D/E298D	1	–	[2]
	C889T/IVS2 + 1G > A	Q297X/Frameshift	1	0, 0, 0	[47]
	G774A/G490A	W258X/G164S	1	0, 0, 0	[4]
	T1289C/+	M430 T/+	1	0, 0, 0	[4]
	937-999del (63 bp)	D313-P333del/+	1	0, 0, 0	[5]
	G774A/T1253G	W258X/L418R	1	0, 0, 0	[6]
	G774A/G371 T	W258X/R124L	1	0, 0, 0	[7]
Korea	G774A/G774A	W258X/W258X	3	1, 2, 0	[28, 48, 49]
	G774A/+	W258X/+	2	0, 0, 1	[28]
	G774A/G1430A	W258X/R477H	1	0, 0, 1	[28]
	G269A /+	R90H/+	1	1, 0, 0	[28]
China	G269A/G269A	R90H/R90H	2	0, 1, 0	[30]
	151delG/+	A51fsX64/+	2	0, 1, 0	[11]
	C233T/A1145T	P78L/Q382L	1	0, 0, 1	[8]
	C650T/+, SLC2A9 mut	T217 M/+, SLC2A9 mut	1	0, 1, 0	[11]
	C650T/C1546A	T217 M/P516T, SLC2A9 mut	1	0, 0, 0	[11]
	G269A/1289_1290insGG	R90H/M430fsX466	1	0, 0, 0	present study
Czech	1245_1253del/1245_1253del	L415_G417del/ L415_G417del	7	–	[12, 50]
	1245_1253del/C1400T	L415_G417del/T467 M	4	0, 1, 0	[12, 50]
	C1400T/C1400T	T467 M/T467 M	2	0, 0, 0	[50]
	G1096C/G1430A	G366R/R477H	2	0, 0, 0	[51]
Iraqi jews	C1216T/C1216T	R406C/R406C	2	0, 0, 0	[52]
	C1216T/+	R406C/+	2	0, 0, 0	[52]
	C1216T/+; G1330A/G1330A	R406C/+; G444R/G444R	2	1, 0, 0	[52]
Israel–Arab	+/+	+/+	4	0, 2, 0	[25]
Italy	+/+	+/+	1	0, 1, 0	[24]
Macedonian and British	C1300T/+	R434C/+	3	1, 0, 0	[9]
	G1301A/+	R434H/+	2	0, 0, 0	[9]
	C1039A/+	R347S/+	1	0, 0, 1	[9]
	G1162A/+	V388 M/+	1	1, 0, 0	[9]
	T224C/+	I75T/+	1	1, 0, 0	[9]
African Americans	G193 T	G65 W	–	–	[10]

+ indicates the wild-type allele. Note: G65 W was identified to be associated with lower SUA level by GWAS, not case reports

^a^according to coding sequence

^b^according to amino acid

In the present study, we confirmed the presence of a heterozygous c.G269A/p.R90H mutation in the patient, and a previous functional experiment revealed that the mutation significantly decreased urate transport activity but it did not affect membrane localization [4]. In our bioinformatics analysis, the c.269G > A mutation was considered “damaging” by Polyphen-2, LRT, and MutationTaster prediction, which further corroborates its pathogenic property. Protein domain analysis showed that URAT1 has 12 TM helices, and we made a discovery that amino acid substitution p.R90H, which is located close to the second TM (TM2), is highly conserved among different species, suggesting the probable functional significance for urate transport. Further, we conducted the molecular modeling to predict the effect of this mutation on protein structure. In the wild model, Arg90 formed hydrogen bonds with Cys88 and Gln93 effectively, building the conformational stability of this intracellular domain. In the mutation model, the hydrogen bond between His90 and Gln93 were weakly formed, which may decrease the stability of this domain and further affect urate transport activity. Of course, this effect may also not be strong enough to impair the domain’s stability. Instead, His90 per se has fewer hydrogen atoms, which can form hydrogen bonds, and thus it may impair the coupling between URAT1 and other unknown proteins that together participate in urate transport. This mutation was identified in the Exome Sequencing Project with the frequency of 7.70*10^− 5^, but it was not found in 1000 Genomes. In a recent study from Japan, the prevalence of p.R90H was found to be 0.28% in healthy examined participants and these subjects showed a marked decrease of SUA level [34]. All of these materials confirm that this mutation is not prevalent in the general population and it is responsible for protein malfunction. We also detected a novel heterozygous mutation of c.1289_1290insGG that resulted in a truncated protein of 466 amino acids from the position 430 (p.M430fsX466), with the amino acid sequence altered compared to the wild mature protein of 553 amino acids. Protein domain analysis showed that this truncated protein lacks the last three transmembrane domains, including the tripeptide motif (S/T)XΦ (X = any amino acid and Φ = hydrophobic residue) at the C-terminal, which interacts with scaffolding protein PDZK1, and together they strengthen the urate transport [22]. Thus, the p.M430fsX466 mutation will possibly lead to weak functioning of urate transport through the disruption of the “transporter complex” formed by URAT1 and PDZK1. This mutation was not identified in either the Exome Sequencing Project or 1000 Genomes, which strengthens the suspected pathogenicity. Taken together, we considered that this patient with such compound heterozygous mutations presents whole functional defects of URAT1 and deficiency of UA reabsorption. Next, we summarized all of the mutations’ frequencies in some open-source databases and bioinformatics prediction results for mutation pathogenicity (see Table 5). All of the mutations were at a very low frequency or not found in the present databases, with most predicted to be damaging or possibly damaging. Combined with our protein analysis for R90H and M430fsX466 mentioned above, we concluded that mutation-induced structural instability or malfunction of the urate transporter complex may be the main mechanisms for this hereditary disorder.

**Table 5:** Mutation frequency and pathogenicity prediction for all *SLC22A12* mutations

Nucleotide change^a^	Amino acid change^b^	Start position (GRCh37.p13 Primary Assembly)	Mutation frequency			Bioinformatics analysis results			
			1000g2015aug_all	ExAC	esp6500siv2_all	SIFT	Polyphen-2	LRT	MutationTaster
151delG	A51fsX64:	Chr11; 64,359,179	.	.	.	.	.	.	.
G193 T	G65 W	Chr11; 64,359,221	.	.	.	D	D	N	N
T224C	I75T	Chr11; 64,359,252	0.0002	0.0002	0.0005	D	D	N	N
C233T	P78L	Chr11; 64,359,261	.	2.51*10^−5^	.	D	D	D	D
G269A	R90H	Chr11; 64,359,297	.	0.0002	7.70*10^−5^	T	D	D	A
G371 T	R124L	Chr11; 64,359,399	.	.	.	T	P	N	N
G412A	V138 M	Chr11; 64,360,260	.	6.59*10^−5^	.	D	D	D	D
G490A	G164S	Chr11; 64,360,338	.	0.0001	.	D	D	N	N
IVS2 + 1 G > A	Frameshift	Chr11; 64,360,355	0.0002	2.48*10^−5^	.	.	.	.	D
C650T	T217 M	Chr11; 64,361,020	.	3.31*10^−5^	.	D	D	N	A
G774A	W258X	Chr11; 64,361,219	0.000998	0.0003	.	.	.	D	A
C889T	Q297X	Chr11; 64,366,046	.	.	.	.	.	N	A
G894 T	E298D	Chr11; 64,366,051	.	.	.	T	D	D	A
937_999del (63 bp)	D313_P333del	Chr11; 64,366,094	.	.	.	.	.	.	.
C1039A	R347S	Chr11; 64,366,364	0.0002	.	.	T	D	D	D
G1082 T	G361 V	Chr11; 64,367,159	.	.	.	T	D	D	A
G1096C	G366R	Chr11; 64,367,173	.	.	.	D	D	D	D
A1145T	Q382L	Chr11; 64,367,222	.	6.72*10^−5^	.	D	D	D	D
G1162A	V388 M	Chr11; 64,367,239	.	0.0002	7.70*10^−5^	T	D	N	N
C1216T	R406C	Chr11; 64,367,293	.	4.23*10^−5^	.	D	D	D	D
1245_1253del	L415_G417del	Chr11; 64,367,322	.	.	.	.	.	.	.
T1253G	L418R	Chr11; 64,367,330	.	1.73*10^−5^	.	D	D	N	A
T1289C	M430 T	Chr11; 64,367,842	.	.	.	T	P	N	D
1289_1290insGG	M430fsX466	Chr11; 64,367,842	.	.	.	.	.	.	.
C1300T	R434C	Chr11; 64,367,853	.	0.0002	7.70*10^−5^	T	P	N	D
G1301A	R434H	Chr11; 64,367,854	0.004593	0.0025	0.0049	D	D	D	D
G1330A	G444R	Chr11; 64,367,883	.	6.07*10^−5^	.	T	D	N	N
C1400T	T467 M	Chr11; 64,368,212	0.001797	0.0014	7.70*10^−5^	T	D	N	N
C1429A	R477S	Chr11; 64,368,241	.	.	.	D	D	D	D
G1430A	R477H	Chr11; 64,368,242	.	9.22*10^−5^	.	D	D	D	D
C1546A	P516T	Chr11; 64,368,358	.	.	.	D	D	D	D
1639_1643del	Frameshift	Chr11; 64,369,000	.	.	.	.	.	.	.

SIFT: D means deleterious, T means tolerated; Polyphen-2: D means probably damaging, P means possibly damaging; LRT: D means deleterious, N means neutral, U means unknown; MutationTaster: A means disease_causing_automatic, D means disease_causing, N means polymorphism. Dot means not found or unknown

^a^according to coding sequence

^b^according to amino acid

* means multiplication sign

Generally, most RHUC patients are asymptomatic with no manifestations of urolithiasis and AKI [1], as with our patient. An inquiry of the patient’s medical history revealed that the woman was a vegetarian who did not favor meat, which contains more purine. Thus, the amount of urate excreted into urine was within the normal range, even if the FEUA was increased, which might explain why our patient was asymptomatic. Treatment is unavailable and outcomes may be severe. So, it is still necessary for the patient to follow the medical orders prescribed by clinicians to prevent the incidence of adverse events. Considering that the offspring of this patient will carry at least one heterozygous mutation and that potential mutations may be located in any of the exons of the *SLC22A12* gene (see Table 5), we recommend the patient’s future husband undergo whole gene sequencing for at least the *SLC22A12* gene, and try to exclude mutant carriers to minimize the risk of severe hyperuricemia in the patient’s offspring.

This study had some limitations. We applied WES to scan the potential variants for the disease, but in each filtering step of the prioritization protocol, there was inevitable bias, which might produce inconsistent results in our validation of the subsequent Sanger sequencing. We analyzed a single patient and our results may not represent the general genetic etiology for Chinese patients with RHUC. In the future, we should conduct a large-scale epidemiological survey in the general population to identify the overall RHUC prevalence in the Chinese population to obtain a full picture regarding the genetic aberrance for this disorder.

In conclusion, we successfully established a Chinese RHUC patient caused by novel compound heterozygous mutations of the *SLC22A12* gene using WES and Sanger sequencing for the first time. As WES is less expensive than traditional methods and more patients are willing to undergo genetic testing, we will acquire a comprehensive mutational landscape for this hereditary disease and further develop specific diagnostic kits for genetic counseling and diagnosis.

## Additional file

Additional file 1: The putative genetic mutations in WES analysis. According to an assumed inheritance of autosomal recessive mode for this disease, we first analyzed the putative genetic mutations homozygous in the patient and heterozygous in both parents. After filtering, 29 variants were retained (see Sheet 1). We also analyzed the putative causal mutations present in the patient but not present in her parents. After filtering, a total of 521 suspicious mutations were screened out (see Sheet 2). (XLSX 298 kb)

## Abbreviations

AKI: Acute kidney injury
BWA: Burrows-Wheeler Aligner
FEUA: Fractional excretion of uric acid
GATK: Genome analysis toolkit
GLUT9: Glucose transporter member 9
GWAS: Genome-wide association studies
LM-PCR: Ligation-mediated polymerase chain reaction
PDZK1: PDZ domain containing 1
RHUC: Renal hypouricemia
SUA: Serum uric acid concentration
TM: Transmembrane
URAT1: Uric acid transporter 1
